# The ovaries of transgender men indicate effects of high dose testosterone on the primordial and early growing follicle pool

**DOI:** 10.1530/RAF-22-0102

**Published:** 2023-04-26

**Authors:** Emily Bailie, Mila Maidarti, Robert Hawthorn, Stuart Jack, Neale Watson, Evelyn E Telfer, Richard A Anderson

**Affiliations:** 1 Institute of Cell Biology, Hugh Robson Building, University of Edinburgh, Edinburgh, UK; 2 Queen Elizabeth University Hospital, Glasgow, UK; 3 Simpson Centre for Reproductive Health, Royal Infirmary, Edinburgh, UK; 4 Spire Thames Valley Hospital, Wexham St, Slough, UK; 5 MRC Centre for Reproductive Health, Queens Medical Research Institute, University of Edinburgh, Edinburgh, UK

**Keywords:** transgender, testosterone, non-growing ovarian follicles, primordial follicles, effect, impact

## Abstract

Androgens are essential in normal ovarian function and follicle health, but hyperandrogenism, as seen in polycystic ovary syndrome, is associated with disordered follicle development. There are few data on the effect of long-term exposure to high levels of testosterone as found in transgender men receiving gender-affirming endocrine therapy. In this study, we investigate the effect of testosterone on the development, morphological health and DNA damage and repair capacity of human ovarian follicles *in vivo* and their survival *in vitro.* Whole ovaries were obtained from transgender men (mean age: 27.6 ± 1.7 years; range: 20–34 years, *n* = 8) at oophorectomy taking pre-operative testosterone therapy. This was compared to cortical biopsies from age-matched healthy women obtained at caesarean section (mean age: 31.8 ± 1.5 years; range: 25–35 years, *n* = 8). Cortical tissues were dissected into fragments and either immediately fixed for histological analysis or cultured for 6 days and subsequently fixed. Follicle classification and morphological health were evaluated from histological sections stained with hematoxylin and eosin and expression of γH2AX as a marker of DNA damage by immunohistochemistry (IHC). In uncultured tissue, testosterone exposure was associated with reduced follicle growth activation, poor follicle health and increased DNA damage. After 6 days of culture, there was enhanced follicle activation compared to the control with further deterioration in morphological health and increased DNA damage. These data indicate that high circulating concentrations of testosterone have effects on the primordial and small-growing follicles of the ovary. These results may have implications for transgender men receiving gender-affirming therapy prior to considering pregnancy or fertility preservation measures.

**Lay summary:**

As part of gender transitioning, transgender men take testosterone therapy. While androgens like testosterone are essential to maintain ovarian health, the effects of long-term testosterone treatment on the ovary are unclear. This study examines whether testosterone impacts ovarian follicle growth activation, follicle health and whether it causes DNA damage. It also looks at how well these follicles grow in tissue culture. The results showed there was a higher proportion of non-growing ovarian follicles in the ovaries of trans men, they appeared less healthy and there were higher levels of DNA damage. After 6 days of tissue culture, there were more growing follicles in transgender ovaries compared to control, but follicle health further deteriorated and there are increased levels of DNA damage. These results identify new effects of testosterone on the ovary and highlight the importance of discussing fertility preservation options prior to starting testosterone.

## Introduction

Reproductive choices are important for transgender individuals, as they are for the general population (De Sutter 2001). In the absence of surgery, trans men retain the potential for pregnancy, and international guidelines recommend consideration of fertility preservation options prior to commencing gender-affirming testosterone therapy (Coleman *et al.* 2012, Anderson *et al.* 2020). Current fertility preservation options available are oocyte and embryo cryopreservation (Mattawanon *et al.* 2018, Anderson *et al.* 2020). However, despite 54% of transgender men expressing a desire to have their own biological children (Wierckx *et al.* 2012), the uptake of fertility preservation interventions is as low as 3% (Birenbaum-Carmeli *et al.* 2021). Ovarian tissue cryopreservation is also potentially of value to the transgender population, with the aim of utilising immature oocytes from the preserved ovarian cortex (De Roo *et al.* 2016). Tissue culture systems have been developed over decades with the goal of producing developmentally competent oocytes (Bertoldo *et al.* 2018, Telfer & Andersen 2021). Complete *in vitro* growth of primordial follicles with subsequent *in vitro* maturation (IVM), IVF and production of live offspring has been achieved in the mouse (Eppig *et al.* 1996, O'Brien *et al.* 2003) and systems that support the development of human immature follicles to antral stages (Telfer *et al.* 2008) and to the development of mature oocytes that can reach Metaphase II have been developed (McLaughlin *et al.* 2018). There are, however, limited data on the effects of long-term gender-affirming testosterone treatment on the ovary.

Androgens are essential in maintaining normal ovulatory function and follicle health (Bertoldo *et al.* 2019). Excessive androgen as demonstrated in the ovaries of females with polycystic ovary syndrome (PCOS) results in an increase in follicular recruitment with subsequent arrest in follicle development and accumulation of pre-antral follicles (Nisenblat & Norman 2009). Along with anovulation, these patients typically have poor ovarian follicle health and subfertility (Dumesic *et al.* 2015). Animal studies have revealed that administering high doses of testosterone results in an ovarian morphology with similarities to PCOS (Murray *et al.* 1998, Vendola *et al.* 1998, Abbott *et al.* 2009). However, the results of studies investigating the effect of exogenous testosterone on human ovaries are variable. The ovaries of transgender men undergoing testosterone therapy have been reported to show increased numbers of antral and atretic follicles, similar to PCOS (Amirikia *et al.* 1986, Futterweit & Deligdisch 1986, Spinder *et al.* 1989, Pache *et al.* 1991, Grynberg *et al.* 2010), whilst others have found a normal follicle distribution (Ikeda *et al.* 2013, De Roo *et al.* 2017).

This study aimed to investigate the effects of long-term exposure to high levels of testosterone on the human ovary. Specifically, to identify if exogenous testosterone therapy impacts the pool of non-growing and early follicles *in vivo*, and the response to *in vitro* culture. The effects on follicle health and growth initiation, markers of DNA damage and its repair were also investigated.

## Materials and methods

### Ovarian cortical tissue

Whole ovaries were obtained with informed consent from transgender patients undergoing hysterectomy and bilateral oophorectomy as part of gender transitioning (mean age: 27.6 ± 1.7 years; range: 20–34 years, *n* = 8). Ethical approval of this study was given by the local ethics committee (ref LREC16/SS/0114). Ovaries were collected in theatre and placed in pre-warmed dissection medium (Leibovitz L-15 supplemented with glutamine (2 mM; Gibco), sodium pyruvate (2 mM), human serum albumin (HSA) (3 mg/mL), penicillin G (75 µg/mL) and streptomycin (50 µg/mL) all from Sigma-Aldrich).

Age-matched contemporaneous control ovarian cortical biopsies (mean age: 31.8 ± 1.5 years; range: 25–35 years, *n* = 8) were obtained from women undergoing elective caesarean section (Telfer *et al.* 2008).

### Tissue preparation and fragment culture

On arrival in the laboratory, ovaries were transferred into fresh, pre-warmed dissection medium and then placed individually on a shallow glass Petri dish. The ovarian cortex was dissected into fragments using a no. 24 scalpel blade and fine forceps to secure the ovary, ensuring to avoid areas containing haemorrhagic or cystic follicles and then placed in fresh pre-warmed dissection medium. Cortical strips including samples from both ovaries were selected at random, fixed in neutral-buffered formalin (NBF) and processed as uncultured controls for histological evaluation of follicle distribution and morphology. Tissue stretching was performed as previously described (McLaughlin *et al.* 2018) to promote *in vitro* activation of follicles and to aid follicle visualisation. Using an angled incision, tissue strips were divided into fragments ∼4 × 2 × 1 mm thick. Fragments were cultured individually in 24-well flat-bottomed plates at 37°C in 300 µL/well culture medium (McCoys 5a medium supplemented with 25 mM HEPES (Gibco), glutamine (3 mM; Gibco), HSA (0.1%), penicillin G (0.1 mg/mL), streptomycin (0.1 mg/mL), transferrin (2.5 µg/mL), selenium (4 ng/mL), human insulin (10 ng/mL), recombinant human FSH (1 ng/mL) and ascorbic acid (50 µg/mL) all from Sigma-Aldrich) for 6 days in humidified air with 5% CO_2_; half the culture medium was removed and replaced with fresh medium on alternate days (days 2 and 4). At the end of the culture period, all fragments were fixed in NBF for histological processing.

### Histological methods and tissue analysis

**Figure 1:**
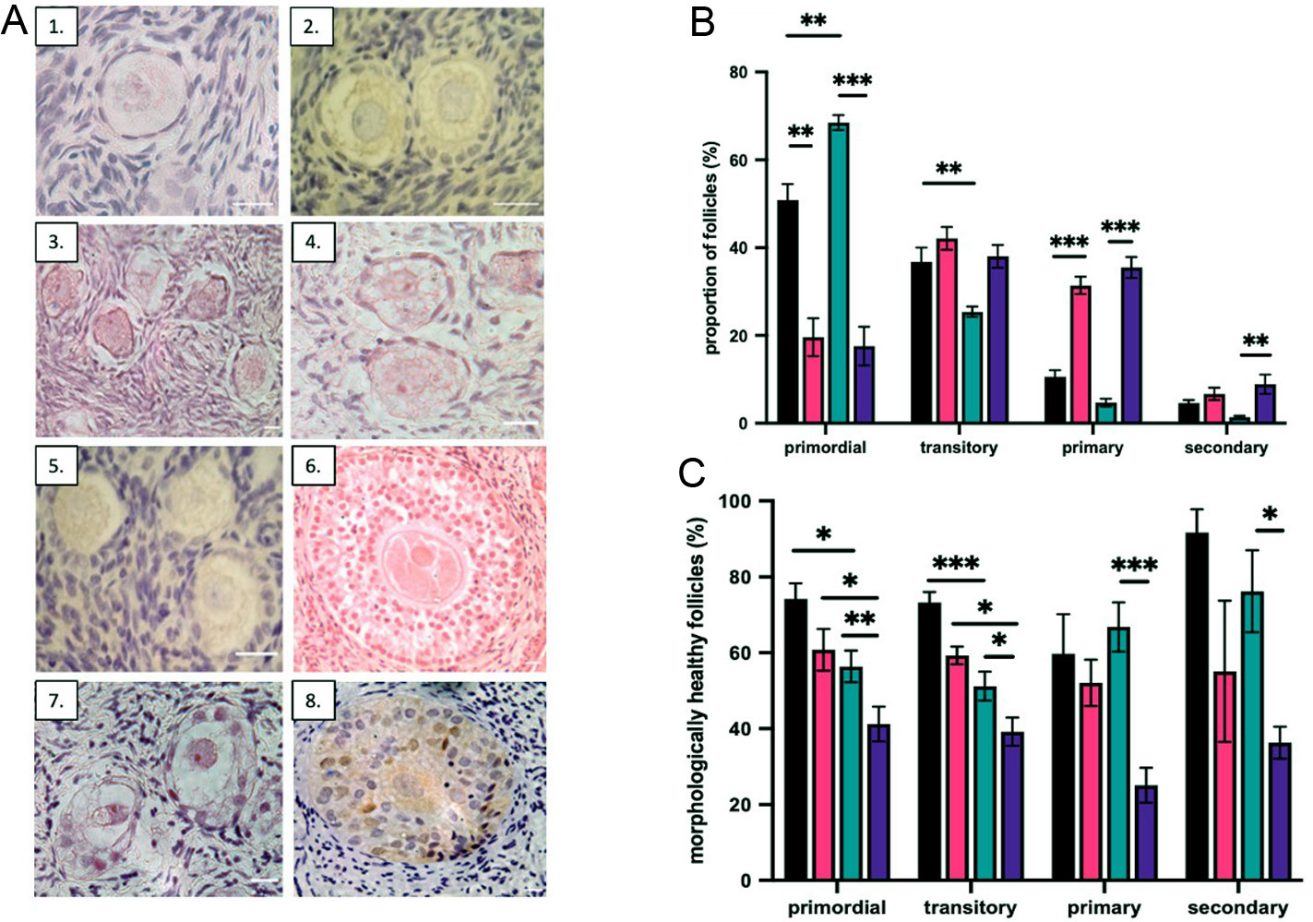
(A) Photomicrographs of non-growing and growing follicles from both groups. (A1) primordial follicle from control tissue, (A2) transitory follicles from control tissue, (A3–4) primordial follicles from transgender tissue, (A5) primary follicles from control tissue, (A6) secondary follicle from control tissue, (A7) primary follicles from transgender tissue and (A8) secondary follicle from transgender tissue. (B) Follicle classification: proportion of follicles in each classification stage in transgender and control tissue at D0 and D6 (*n* = 8 patients per group). In the analysis using Kruskal–Wallis test with *post hoc* Dunn test, results were analysed per patient and expressed as mean± s.e.m., *P*-value < 0.05 was considered significant, ***P ≤ 0.001, **P ≤ 0.01, *P < 0.05. Black bars control D0, pink bars control D6, green bars transgender D0 and purple bars transgender D6. (C) Morphological health: proportion of morphologically healthy follicles at each follicle stage in control and transgender tissue D0 and D6 (*n* = 8 patients per group). In the analysis performed using Kruskal–Wallis test with *post-hoc* Dunn test, results were analysed per patient and expressed as mean ± s.e.m., *P*-value < 0.05 was considered significant, ***P ≤ 0.001, **P ≤ 0.01, *P < 0.05. Black bars control D0, pink bars control D6, green bars transgender D0 and purple bars transgender D6.

After fixation for 24 h, individual fragments were dehydrated through graded alcohols and cleared in cedar wood oil before being embedded in paraffin wax and serially sectioned at 6 μm thickness. Tissue sections were stained with hematoxylin and eosin and every 10th section was analysed using a light microscope under 40× magnification. Follicles were classified using a modification of an established system: primordial follicle (oocyte surrounded by a complete or incomplete single layer of flattened granulosa cells), transitory follicle (an oocyte surrounded by a mixed layer of flattened and cuboidal granulosa cells), primary follicle (oocyte surrounded by a single layer of cuboidal granulosa cells) and secondary follicle (an oocyte surrounded by two or more complete layers of cuboidal granulosa cells) (Pedersen & Peters 1968). For the main analysis of the effect of culture, primordial and transitory follicles were distinguished and analysed separately. For the DNA damage and repair immunohistochemical analyses, they were combined and analysed as non-growing follicles (Gougeon & Chainy 1987 *b*,Westergaard *et al.* 2007). The number and developmental stage of follicles from D0 and cultured D6 tissue were recorded per patient. To prevent double counting, only follicles with an oocyte nucleus were analysed. The classification of healthy follicles was based on the same criteria as previously described (Telfer *et al.* 2008, McLaughlin *et al.* 2014). In brief, for follicles to be categorised as morphologically normal, the oocyte must be grossly circular, surrounded by a zona pellucida and have <10% of pyknotic granulosa cells present (Fig. 1A).

### Immunofluorescence

Immunofluorescence was used to identify and quantify the presence of γH2AX, a marker of DNA damage, in oocytes and granulosa cells of ovarian follicles from both transgender and control D0 and D6 tissue. Tissue sections were dewaxed, rehydrated and immersed in phosphate-buffered saline (PBS) with 0.1% (v/v) Triton X-100 (PBST) (pH 7.2–7.4). Antigen retrieval in 10 mM sodium citrate (pH 6.0) was performed by microwaving slides followed by cooling at room temperature. After washing, slides were incubated for 1 h at room temperature with blocking solution (5% goat serum in PBST) and then probed with primary antibody γH2AX (NB100-384, 1:1000; Novusbio, Centennial, CO, USA) overnight at 4°C. Blocking solution without primary antibody acted as a negative control. Next, the slides were washed and incubated with a secondary antibody (Cy3-conjugated affinity pure donkey anti-rabbit IgG (H + L), 1:250; Jackson Laboratories) for 1 h. Following further PBST washes, the sections were mounted in a Vectashield hardset with 4′-6-diamidino-2-phenylindole (DAPI) (H-1500; Vector Laboratories, Peterborough, UK).

**Figure 2:**
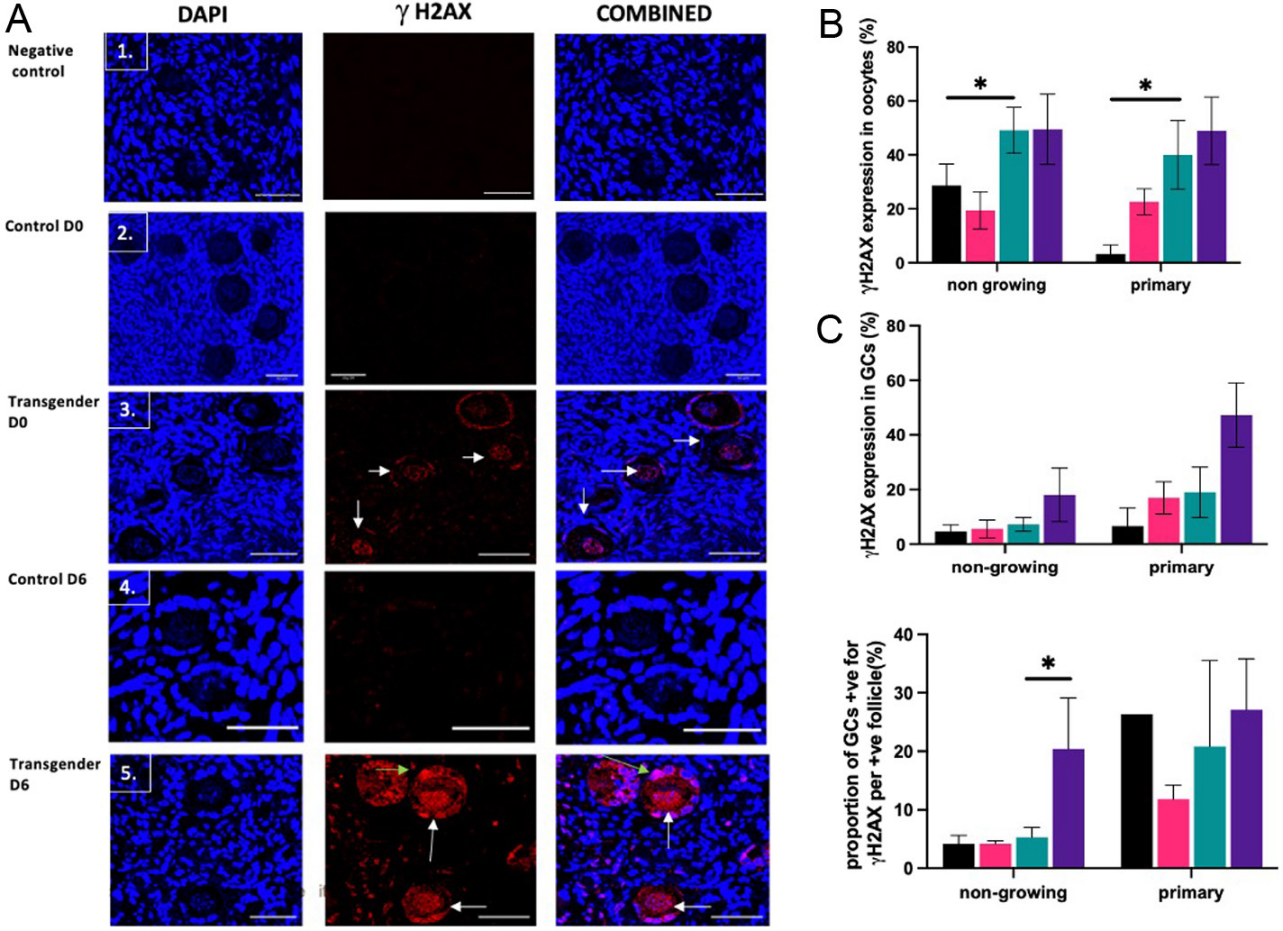
(A) Localisation of γH2AX by immunofluorescence in human ovarian tissue. γH2AX (red) and DAPI (blue) staining in oocyte and granulosa cells. γH2AX staining is identified as red foci within nuclei (white arrows) in oocytes and granulosa cells (green arrows). (1) negative control, (2) control D0 – non-growing follicles, oocytes and granulosa cells negative for γH2AX, (3) transgender D0 – foci of γH2AX identified within the oocytes and granulosa cells of non-growing follicles, (4) control D6 – growing follicles negative staining for γH2AX, (5) transgender D6 – growing follicles positive foci identified within oocytes and granulosa cells. Scale bar = 40 μm. (B–C) Positive expression of γH2AX in oocytes (B) and granulosa cells (C) (total number of follicles *n* = 840, control D0 *n* = 122, transgender D0 *n* = 384, control day 6 *n* = 145, transgender day 6 *n* = 189). Expression of γH2AX in oocytes and granulosa cells was analysed per patient (*n* = 8 patients per group) and results are expressed as the mean ± s.e.m. Expression in oocytes was analysed using the Kruskal–Wallis test with the *post hoc* Dunn test. Expression of γH2AX in granulosa cells and proportion of granulosa cells in affected follicles were analysed using the Kruskal–Wallis test followed by the *post hoc* Dunn test. Statistical significance assigned *P* < 0.05. *P < 0.05. Black bars control D0, pink bars control D6, green bars transgender D0 and purple bars transgender D6.

Images were captured using a Zeiss LSM 800 confocal microscope with ×20 magnification (Fig. 2A). Five sections were analysed per patient and images were taken of every follicle with an oocyte nucleus present and analysed using ImageJ. The proportion of oocytes with positive expression of γH2AX was calculated per patient and analysed as mean ± s.e.m. For the granulosa cells, the proportion of follicles with any positive expression of primary antibody within the granulosa cells was calculated per patient followed by calculating the proportion of positive granulosa cells per total number of granulosa cells per positively stained follicle. Both results were analysed as the mean ± s.e.m. ImageJ was used to quantify the intensity of γH2AX immunofluorescence staining in both oocytes and granulosa cells. This was carried out by measuring the mean grey value. The results were analysed per patient and presented as the mean ± s.e.m.

### Immunohistochemistry

DNA repair proteins were localised in tissue sections from both control and transgender ovarian tissue at D0 and D6 using antibodies against Rad51 (137323; 1:500; Abcam), Ataxia-Telangiesctasia Mutated (ATM) (ab78; 1:500; Abcam) and meiotic recombination (MRE) 11 (NB100-142; 1:1000; Novusbio). Antigen retrieval was performed as described earlier, then slides were immersed in 3% (v/v) hydrogen peroxide to quench endogenous peroxidase activity. The slides were washed and incubated for 1 h in appropriate blocking solution (150 μL goat serum in 10 mL PBST) followed by incubation in suitably diluted primary antibodies overnight at 4°C. Blocking solution without primary antibody acted aa a negative control.

After washing, sections were incubated for 30 min with biotinylated secondary antibody at room temperature (Vector Laboratories) and then processed using an ABC kit as per manufacturer instructions (Vectastain Elite ABC kit; Vector Laboratories). DAB (3,3′-diaminobenzidine) peroxidase substrate kit (Vector Laboratories) solution was applied to the sections and then counterstained with haematoxylin. Five sections were analysed per patient. The proportion of oocytes with positive expression of a DNA repair protein was calculated per patient and analysed as the mean ± s.e.m. For the analysis of granulosa cells, the proportion of follicles with any positive expression of primary antibody within the granulosa cells was calculated per patient followed by calculating the proportion of positive granulosa cells per total number of granulosa cells per positively stained follicle.

### Statistical analysis

All data were analysed using SPSS statistical software version 24 (SPSS, Inc.). Graphs were generated using GraphPad software version 7 (Graphpad Inc.). All results are presented as mean ± s.e.m. Given the potential for large interpatient variability, non-parametric Kruskal–Wallis test with Dunn *post hoc* test was used for all statistical analyses. Statistical significance was assigned as *P* < 0.05.

## Results

### Patient characteristics

In total, tissue from eight trans men who underwent a hysterectomy and bilateral oophorectomy (mean age 27.6 ± 1.7 years; range 20–34 years, BMI 27.5 ± 1.4) was included in this study. Pre-operative measurement of reproductive hormones (Table 1) showed values in the normal male range. All patients had been treated with 1000 mg of testosterone undecanoate at approximately 12-week intervals prior to tissue collection (range of treatment 18 months–10 years) and were non- or ex-smokers with no significant past medical history, in particular, no history of PCOS (Supplementary Table 1, see section on supplementary materials given at the end of this article).

**Table 1:** Overview of patient blood results (*n* = 5, patients who consented to hormone analysis). Samples were assayed as part of normal care in a hospital laboratory.

Variable	Mean	s.e.m.	Range	Reference range
Age, years	27.6	2.32	20–34	
FSH, µ/L	4.06	0.4	2.9–4.9	1–10
LH, µ/L	3.7	1.7	0.5–9.6	1–9
Oestradiol, pmol/L	122.8	16.3	63–156	0–160
Testosterone, nmol/L	15.5	0.84	12.3–17.2	10–38

### Testosterone exposure and follicle distribution and morphological health

On D0, 32 cortical fragments were obtained, 16 from 8 transgender patients and 16 from 8 control patients, to determine follicle classification and morphological health. As more tissue sections were available from the transgender tissue, more follicles were analysed, transgender 3871 follicles, control 655 follicles, totalling 4526 follicles (Fig. 1A). At D0, the most prevalent follicle type in both groups was primordial, with a significant higher proportion in transgender tissue compared to the age-matched control (67.4 ± 1.7% vs 50.9 ± 1.5% *P* < 0.005) (Fig. 1B). There was a smaller proportion of transitory follicles in the transgender tissue (26.5 ± 1.2% vs 33.9 ± 3.2%, *P* < 0.005). Differences in the proportion of primary (4.7 ± 0.9% vs 10.6 ± 1.5%, *P* = 0.054) and secondary (1.4 ± 0.4% vs 4.6 ± 0.7%, *P* = 0.2) follicles did not reach statistical significance, and no pre-antral or antral follicles were identified in either group.

At D0, the proportion of morphologically healthy primordial and transitory follicles was significantly lower in the transgender tissue compared to the control (primordial: 56.4 ± 4.2% vs 72.1 ± 4.2%, *P* ≤ 0.05, transitory: 51.2 ± 3.8% vs 73.3 ± 2.7%, *P* ≤ 0.001 (Fig. 1C). There were no differences in morphological health of primary and secondary follicles between groups at D0 (primary: 66.8 ± 2.8% vs 59.7 ± 4.6%, *P* = 0.9, secondary: 76.2 ± 10.8% vs 91.7 ± 8.3%, *P* = 0.78).

### Testosterone exposure and follicle activation and survival *in vitro*

On D6, 32 cortical fragments were obtained, 16 from 8 transgender patients and 16 from 8 control patients, to determine the follicle classification and morphological health of follicles after 6 days of culture with a total of 3367 follicles (transgender: 2617 follicles, control: 750 follicles) analysed (Fig. 1A). The proportion of primordial follicles was significantly lower at D6 compared to D0 in both groups (transgender 57.5 ± 2.8% vs 17.6 ± 4.4, *P* < 0.001, control 50.9 ± 3.6% vs 19.6 ± 4.3%, *P* < 0.005 (Fig. 1B) with no change in the proportion of transitory follicles. Thus, the population of non-growing follicles after 6 days in culture was lower in the transgender group than in controls (55.6 ± 3.3% vs 61.7 ± 2.4%, *P* < 0.005). In both groups, this decline was balanced with a reciprocal increase in the proportion of growing follicles, in particular primary follicles (transgender: primary 4.7 ± 0.9% to 35.5 ± 1.9%, *P* < 0.001, secondary 1.4 ± 0.4% to 8.5 ± 2.3%, *P* < 0.005, control: primary 10.6 ± 1.5% to 32.5 ± 1.7%, *P* < 0.001, secondary 4.6 ± 0.7% to 8.5 ± 2.3%, *P* = 0.99).

On D6 in the transgender tissue, there was a reduction in the proportion of morphologically healthy follicles across all follicle types from D0 (primordial 56.4 ± 4.2% to 41.2 ± 4.6%, transitory 51.2 ± 3.8% to 39.2 ± 3.7%, primary 66.8 ± 6.5% to 25.1 ± 4.6%, secondary 76.2 ± 10.8% to 36.3 ± 4.2%, *P* < 0.05 for all follicle types) (Fig. 1C). In contrast, in control tissue, on day 6, the morphological health of follicles remained stable across all follicle types (primordial: 74.2 ± 4.1% to 60.8 ± 5.5%, *P* = 0.71, transitory: 73.3 ± 2.7% to 59.3 ± 3.3%, *P* = 0.40, primary: 59.6 ± 10.4% to 52.1 ± 2.1%, *P* = 0.88, secondary 91.7 ± 8.3% to 55.1 ± 18.6%, *P* = 0.21).

### Testosterone exposure and DNA damage and DNA double-strand breaks repair capacity in follicles

To identify whether testosterone exposure was associated with DNA damage, five sections per patient from both transgender and control groups were used to identify expression of γH2AX as a marker of DNA damage in oocytes and the granulosa cells of ovarian follicles (Fig. 2A 1–5). Both D0 and D6 tissue was analysed and included 840 follicles (transgender 573 follicles, control 267 follicles).

At D0, the proportion of oocytes from non-growing and primary follicles expressing γH2AX was significantly higher in transgender compared to control tissue (non-growing: transgender 49.1 ± 8.5% vs control 28.6 ± 8%, primary 40 ± 12.7% vs 3.3 ± 3.3%, both *P* < 0.05) (Fig. 2B). Insufficient numbers of secondary follicles were identified for analysis.

The proportion of non-growing follicles expressing γH2AX in the granulosa cells at D0 did not significantly differ between groups (Fig. 2C), with comparable proportions of granulosa cells per follicle affected (Fig. 2C). In primary follicles, a higher proportion of follicles expressed γH2AX in the granulosa cells in the transgender tissue compared to control (19 ± 9.2% vs 6.6 ±6.7%, *P* < 0.05) but with comparable proportions of granulosa cells per follicle affected (20.8 ± 14.7% vs 26.3 ± 2.1%, *P* = 0.67).

On D6, expression of γH2AX in the oocytes of non-growing follicles remained unchanged in both groups (Fig. 2B). Expression of γH2AX remained high in primary oocytes in transgender tissue (40 ± 12.7% to 48.9 ± 12.5%, *P* = 0.5); while there appeared to be an increase in control, this was variable and not statistically significant 3.3 ± 3.3% to 22.6 ± 4.8%, *P* = 0.5).

At D6, in the transgender non-growing follicles, there was a significant increase in the percentage of granulosa cells positive for γH2AX per follicle (5.4 ± 1.7% to 20.4 ± 8.7%, *P* < 0.05), whereas γH2AX expression remained at low levels in control tissue follicles (Fig. 2C). In primary follicles, there was no significant change in the proportion of follicles expressing γH2AX in the granulosa cells nor the percentage of granulosa cells positive for γH2AX per follicle in both groups (Fig. 2C).

No significant differences in the intensity of staining in oocytes and granulosa cells were detected between control and transgender tissue across all follicle types at D0 and D6.

**Figure 3:**
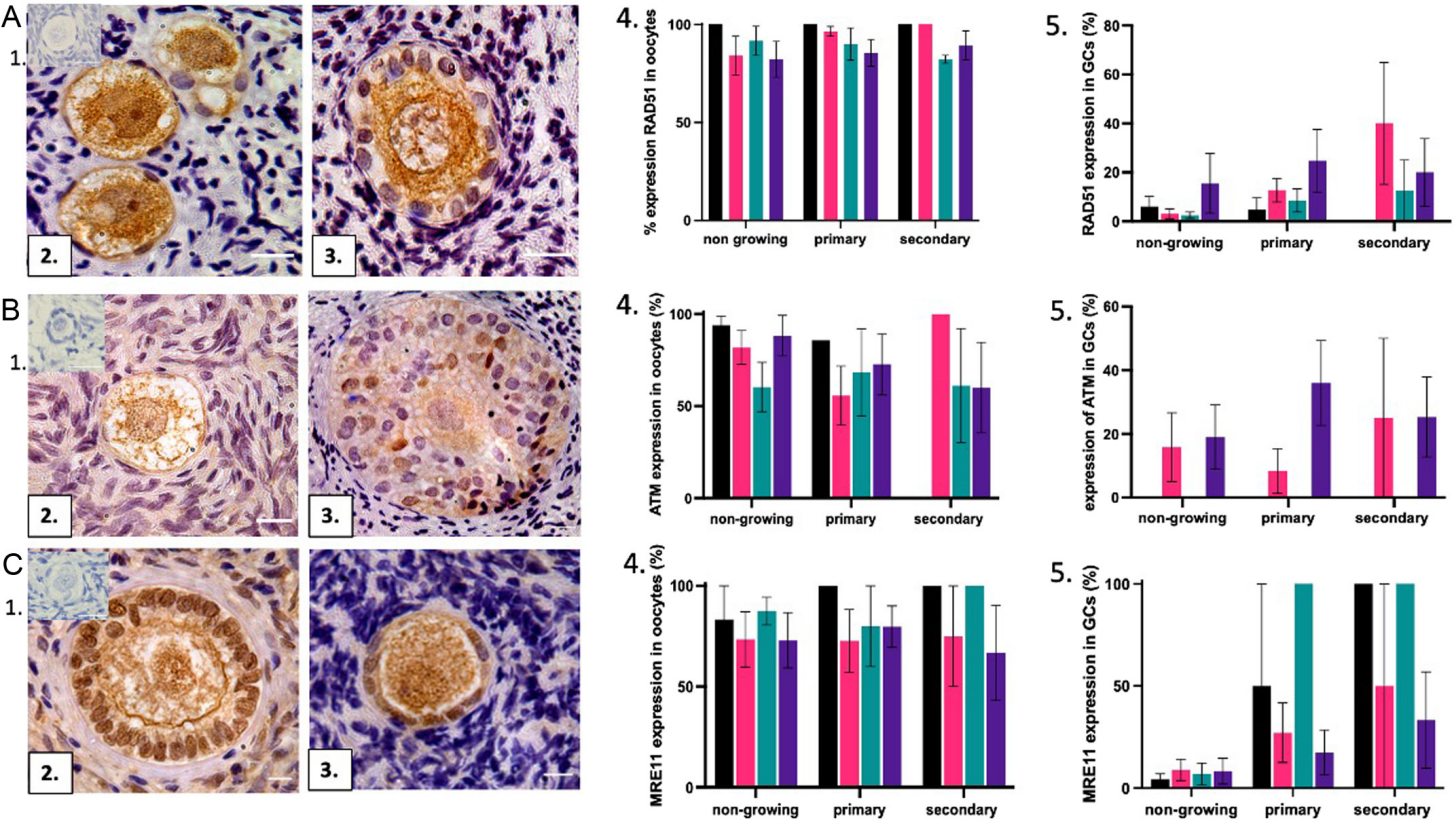
Immunohistochemical detection of RAD51, ATM and MRE11. Photomicrographs of ovarian follicles for RAD51 (A1–3), ATM (B1–3) and MRE11 (C1–3). Negative control (1), control follicles A2/B2/C2, transgender follicles A3/B3/C3. Positive staining (brown) in oocytes in A2–3, B2–3 and C2–3. Positive staining in granulosa cells in B3 and C2–3. Scale bar = 40 µm. Graphs showing positive expression in oocytes (A4, B4, C4) and granulosa cells (A5, B5, C5) (total number of follicles, RAD51 *n* = 834, ATM *n* = 800, MRE11 *n* = 550, *n* = 8 patients per group). Results were analysed per patient and expressed as the mean ± s.e.m. Expression in oocytes was analysed using Kruskal–Wallis test with the *post hoc* Dunn test. Expression in granulosa cells was analysed using Kruskal–Wallis test with the *post hoc* Dunn test. Statistical significance assigned *P* < 0.05. Black bars control D0, pink bars control D6, green bars transgender D0 and purple bars transgender D6.

To identify whether testosterone affects the DNA repair capacity of ovarian follicles, expression of the DNA repair proteins ATM, RAD51 and MRE11 which are involved in the process of homologous recombination was analysed (Fig. 3A1–3/B1–3/C1–3). Five sections per patient from both control and transgender groups were analysed for expression in both oocytes and granulosa cells.

At D0, there was no significant difference in expression of RAD51, ATM and MRE11 in oocytes and granulosa cells between transgender and control follicles (*P* > 0.05) (Fig. 3). At D0, RAD51, ATM and MRE11 were found in a high proportion of both transgender and control oocytes across all follicle types (Fig. 3 A4/B4/C4). In both groups, RAD51 and MRE11 were expressed in a low proportion of granulosa cells in non-growing and primary follicles, with no expression of ATM identified (Fig. 3 A5/B5/C5).

At D6, there was no significant difference in the expression of RAD51, ATM and MRE11 in oocytes and granulosa cells between groups (*P* > 0.05). At D6, in both groups, expression of RAD51 remained high in oocytes and low in granulosa cells across all follicle types (Fig. 3A4 and Fig. 3A5). Expression of ATM in oocytes in both groups was comparable to D0 across all follicle types (Fig. 3B4). ATM was detected in variable levels in granulosa cells in all follicle types in both groups (Fig. 3A5). In both groups, levels of MRE11 expression remained high in oocytes at D6 (Fig. 3C4) with more variable expression of MRE11 in granulosa cells, particularly in growing follicles (Fig. 3C5).

## Discussion

In this study, we have compared the distribution of follicle stages and their health in ovaries exposed to high levels of testosterone in trans men compared to controls. The effect of *in vitro* culture on follicle distribution and health was also examined, together with an analysis of markers of DNA damage and its repair. A significantly higher proportion of primordial follicles was found in transgender tissue compared with similarly aged controls, and these follicles had poorer morphological health and increased levels of DNA damage.

An increased proportion of primordial follicles in transgender ovarian tissue compared to control suggests that testosterone therapy has a suppressive effect on follicle activation *in vivo*. Similar proportions of primordial follicles were found in other studies (De Roo *et al.* 2017, Borrás *et al.* 2021), but our study found a smaller proportion of primary follicles. These studies concluded that testosterone-exposed ovaries had a similar cortical follicle distribution to cisgender females. However, those studies did not use contemporaneous controls and used historic data from the literature (Gougeon & Chainy 1987 *a*) where the age range was 19–49 or based on data from cisgender females who were known to be infertile (Lass *et al.* 1997). This study used closely aged women who donated ovarian cortical tissue at the time of caesarean section with samples analysed at the same time by the same observer. In pregnancy, follicle-stimulating hormone (FSH) and luteinizing hormone (LH) levels are persistently low for the duration of pregnancy as a result of the inhibitory effects of high levels of circulating oestradiol and progesterone on the hypothalamus and pituitary, suppressing ovulation (Choi & Smitz 2014, Stilley & Segaloff 2018). However, the process of follicle recruitment and early growth is gonadotrophin-independent; therefore, pregnancy is likely to have no effect on this process (Hsueh *et al.* 2015, Zhang *et al.* 2023). In transgender men taking gender-affirming endocrine therapy, FSH and LH levels are also low or similar to the follicular phase of the menstrual cycle in cisgender women (De Roo *et al.* 2017, Greene *et al.* 2021) with low levels of oestradiol (Chan *et al.* 2018). In this study, testosterone levels were within the male range, without marked gonadotrophin suppression.

The choice of appropriate controls is important for these studies, as accessing tissue from healthy, aged matched non-pregnant women is rarely possible. The use here of samples from pregnant cisgender females has advantages as they are fertile healthy samples, and additionally, the high steroid/low gonadotrophin environment has parallels to gender-affirming treatment but with female testosterone levels. However, the effect of pregnancy on follicle DNA damage and capacity for repair is unknown. Using tissue from patients with ovarian pathologies equally has its own limitations: a study of follicle distribution and ovarian reserve in patients with a range of both benign and malignant ovarian pathologies concluded that the cortex surrounding an ovarian malignancy has reduced follicle density, and in women with benign ovarian lesions, stromal proliferation was observed (Pavone *et al.* 2014). Similarly, the ovarian cortex from women with an endometrioma had a reduced follicle number, poorer morphological health and fibrotic change within the stroma (Maneschi *et al.* 1993).

There is limited information regarding the long-term impact of testosterone on ovarian follicles. Reassuringly, after cessation of testosterone therapy, many transgender men successfully conceive naturally (Light *et al.* 2014) or through artificial reproductive techniques (Adeleye *et al.* 2019, Leung *et al.* 2019). Testosterone has been reported to have marked effects on ovarian morphology with abnormalities including stromal hyperplasia, thickened ovarian cortex and polycystic ovary morphology (Futterweit & Deligdisch 1986, Spinder *et al.* 1989, Grynberg *et al.* 2010, Ikeda *et al.* 2013). Testosterone therapy does not appear to deplete the primordial follicle pool from the ovarian cortex of transgender men (Van Den Broecke *et al.* 2001) or affect the *in vitro* maturation potential of cumulus–oocyte complexes (COCs) harvested from the ovarian medulla (De Roo *et al.* 2017, Lierman *et al.* 2017). It has however been reported to enhance follicle activation in the mouse ovary through the nuclear exclusion of Forkhead box O3X (FOXO3A) (Yang *et al.* 2010) although others have found that androgen receptors are not expressed by primordial follicles (Gervásio *et al.* 2014, Walters *et al.* 2018), The dense, fibrous nature of normal human ovarian cortical tissue contributes to suppression of primordial follicle growth, thus preventing mass and premature activation, which would result in early depletion (Silber *et al.* 2018). The ovarian cortex in transgender tissue has been found to be stiffer in comparison to tissue from cancer patients (De Roo *et al.* 2019). This increased resting tissue rigidity may enhance the suppression of residing primordial follicles and be an explanation for the finding here of reduced follicle activation *in vivo*.

Following 6 days of culture, the proportion of non-growing follicles in transgender tissue was significantly lower than in controls, despite being higher before culture. This suggests that the rate of activation of non-growing follicles *in vitro* is higher than in controls. The process of tissue dissection mechanically loosens the ovarian cortex and reduces intrinsic tissue pressure, increasing follicle activation (Smitz & Cortvrindt 2002, Telfer *et al.* 2008, McLaughlin *et al.* 2018, Grosbois & Demeestere 2018). This may have had a greater effect on the testosterone-exposed tissue as accelerated activation can have a detrimental impact on follicle quality (Smitz & Cortvrindt 2002, McLaughlin *et al.* 2014). In studies where primordial follicle activation was enhanced by suppressing phosphatase and tensin homolog deleted on chromosome 10 (PTEN) using Dipotassium bisperoxo oxovanadate (V) (bpV(HOpic)), an increase in the proportion of growing follicles was accompanied by a significant reduction in morphological health in both human (McLaughlin *et al.* 2014, Maidarti *et al.* 2019) and bovine ovarian follicles (Maidarti *et al.* 2019). In this study, with increased follicle activation in cultured transgender ovarian tissue, there was a significant decline in the morphological health of growing follicles compared to the control. This highlights that further optimisation of this stage of the culture process is needed to control follicle activation to ultimately produce a population of high-quality oocytes (Telfer & Zelinski 2013).

γH2AX is a DNA repair protein that binds to the location of DNA damage and controls recruitment of DNA repair proteins (Winship *et al.* 2018). In this study, the proportion of oocytes expressing γH2AX in uncultured D0 non-growing follicles was significantly higher in the transgender group compared to the control, consistent with the histological findings of reduced follicle health. After 6 days of culture, levels of γH2AX in the non-growing and primary follicles from transgender tissue remained stable. The cause of increased DNA damage in testosterone-exposed follicles is not clear, but studies that have used an experimental hyperandrogenic model in culture (Bertoldo *et al.* 2019) found increased levels of reactive oxidative species (ROS) resulting in DNA damage. Oxidative stress is a state whereby ROS outbalances anti-oxidant levels, resulting ultimately in DNA damage and/or cell apoptosis (Lee *et al.* 2022). There is also an increasing body of literature on the role of ROS in the pathogenesis of PCOS (Karadeniz *et al.* 2008, Zhang *et al.* 2008). Increased levels of biochemical markers of oxidative stress have been reported in women with PCOS compared to controls (Sabuncu *et al.* 2001, Palacio *et al.* 2006, Murri *et al.* 2013) whilst others have found variable findings when looking for markers for anti-oxidative stress levels (Sabuncu *et al.* 2001, Zhang *et al.* 2008, Seleem *et al.* 2014). Given that similar histological findings have been identified in both transgender male ovaries and females with PCOS, for example stromal hyperplasia, tunica albuginea thickening (Baba *et al.* 2007), it is possible that ROS could be responsible for the increased DNA damage seen in testosterone exposed ovarian follicles.

It is essential that primordial follicles have a robust system of DNA damage recognition and repair mechanisms to ensure genetic integrity. Unrepaired or incorrectly repaired DNA double-strand breaks (DSBs) result in infertility, miscarriage and genetic defects in offspring (Stringer *et al.* 2018). The preferred mechanism of DNA repair in primordial follicles is homologous recombination (HR), a process of repair which uses the sister chromatid as a template for error-free repair (Winship *et al.* 2018). The process of HR is initiated by the MRN complex consisting of Meiotic recombination 11 (MRE11)-Rad50, Nijmegen breakage syndrome 1 (NBS1), recognising the DNA DSB (Maidarti *et al.* 2020). The binding of the MRN complex to the DNA DSBs allows for the interaction of the NBS1 protein with ATM, resulting in the autophosphorylation of ATM at a serine residue (Rein & Stracker 2014). Following this, ATM phosphorylates H2AX at the C-terminal serine 139 (γH2AX) (Nguyen *et al.* 2021). γH2AX binds to the DNA DSB, resulting in the initiation of the downstream pathway whereby DNA is either repaired or apoptosis occurs (Oktay *et al.* 2015). The DNA repair proteins analysed here are key players within the process of HR.

Similar levels of expression of DNA repair proteins in oocytes were found between the transgender and control groups despite differing levels of expression of γH2AX and morphological health. Small numbers of subjects and follicles being included in the analysis may explain the lack of statistical significance in these experiments. However, a lack of increased expression of DNA repair proteins with increased levels of DNA damage in transgender tissue could indicate suboptimal DNA repair protein recruitment. Ineffective DNA repair is pathognomonic in reproductive aging, resulting in accumulating DNA DSBs (Karanjawala & Lieber 2004, Gorbunova *et al.* 2007, Winship *et al.* 2018). DSBs have been found to accumulate in ovarian follicles of aging mice, with the downregulation of key DNA repair proteins (Oktay *et al.* 2010, Titus *et al.* 2013). It is possible that testosterone may impair the DNA repair capacity of ovarian follicles, resulting in compromised genetic integrity and reduced oocyte quality. However, this study only looks at the first step of follicle growth; therefore, the results of these experiments cannot take into consideration the potential for DNA repair at later stages. This would however align with findings that when COCs harvested from the ovarian medulla of transgender men on testosterone therapy were fertilised, there were significantly impaired fertilisation rates and embryo development (Lierman *et al.* 2021).

A limitation of this study is that there is a discrepancy in the volume of tissue available between groups, with whole ovaries collected from transgender patients and small fragments of ovarian cortex from control subjects. Unlike in rodent studies where absolute follicle counts can be achieved, as often in human studies where biopsies of the ovarian cortex are obtained, the proportion of follicle classification is calculated. Within human cortical tissue, the distribution of follicles can vary significantly between ovarian cortical fragments from the same ovary (Schmidt *et al.* 2003). This presents a difficulty in assessing primordial follicle activation through classifying and counting follicles in uncultured and cultured tissue (Telfer & Zelinski 2013). It is not possible to follow follicle activation in real time, therefore, to comment on follicle activation through assessing different ovarian cortical fragments pre and post culture is difficult.

In this study, there was a wide range of duration of testosterone treatment (range 18 months–10 years). We found no relationship between the markers of DNA damage and duration of treatment (not shown), but it is possible that such a relationship exists and would be identified in a larger study focusing on that question.

In summary, the results from this study indicate that testosterone exposure *in vivo* leads to reduced follicle growth activation, and a reduction in morphological health with apparent DNA damage, with further deterioration after 6 days of culture compared to our control population. These results highlight that further studies are needed to evaluate the long-term sequelae of testosterone on ovarian follicles to guide discussion regarding future fertility and the potential value of fertility preservation in trans men before initiating testosterone treatment. They also indicate a need for further optimisation of the culture system and for customisation depending on the source of ovarian tissue being cultured.

## Supplementary Material

Supplementary figure 1- table of transgender patients’ demographics.

## Declaration of interest

The authors declare that there is no conflict of interest that could be perceived as prejudicing the impartiality of the research reported.

## Funding

This study was funded by MRC grant MR/R003246/1 and Wellcome Trust Collaborative Award in Science: 215625/Z/19/Z.

## Author contribution statement

EB contributed to the conception and design of study, experimental work and data acquisition, analysis and interpretation, and manuscript preparation. MM contributed to the experimental work and data acquisition, analysis and interpretation, and final approval of manuscript. EET contributed to the conception and design of study, data analysis and editing and final approval of manuscript. RAA contributed to the conception and design of study, data analysis and editing and final approval of manuscript. Other authors contributed to the provision of ovarian tissue.
