# Supplementary material for: The ovaries of transgender men indicate effects of high dose testosterone on the primordial and early growing follicle pool
Source: Reprod Fertil. 2023 Apr 26;4(2):e220102. doi: 10.1530/RAF-22-0102 (PMC10160535; doi:10.1530/RAF-22-0102)
Supplement: Supplementary figure 1- table of transgender patients’ demographics. [file supplementary_figure_1.pdf]

Patient	Age	BMI	Testosterone treatment	Frequency of treatment	Duration of treatment (years)
1	25	29.8	Testosterone undecanoate 1000mg	3mthly	5
2	34	26.7	Testosterone undecanoate 1000mg	3mthly	8
3	27	30.8	Testosterone undecanoate 1000mg	3mthly	4
4	32	31.0	Testosterone undecanoate 1000mg	3mthly	10
5	30	29.3	Testosterone undecanoate 1000mg	3mthly	7
6	23	19.8	Testosterone undecanoate 1000mg	3mthly	1.5
7	30	23.0	Testosterone undecanoate 1000mg	3mthly	4
8	20	29.7	Testosterone undecanoate 1000mg	3mthly	3.25

Supplementary figure 1- table of transgender patients' demographics.
